# Draughts in Hospital Wards

**Published:** 1901-04-20

**Authors:** 


### EDITOR'S LETTER-BOX.

### DRAUGHTS IN HOSPITAL WARDS.

To the Editor of *The Hospital.*

Qui Sciat writes: Can nothing be done to alleviate the severe additional sufferings very frequently caused to those who, in some cases, are already suffering acutely by the continual and excessive draughts which seem to be considered essential to sanitation in hospital wards?

That ample ventilation is needful no one, I suppose, will dispute; and experience shows that, in far the greater number of cases, patients *do* recover from the sufferings they undergo through the terrible streams of freezing air poured over them day and night; but it occurs to me that is no excuse for causing them, not unfrequently, weeks of acute pain and needless loss of vital power by a state of things which I am confident no doctor, or matron, or other official would be able to endure quietly as a patient; and unless my senses are no use to me, I have known cases in which this craze for freezing air has resulted in fatal consequences to patients. I write without prejudice: I believe it to be a craze which twenty years hence will be looked back upon as a memory of barbarism.

